# Pain rates in general population for the period 1991–2015 and 10-years prediction: results from a multi-continent age-period-cohort analysis

**DOI:** 10.1186/s10194-020-01108-3

**Published:** 2020-05-13

**Authors:** Davide Guido, Matilde Leonardi, Blanca Mellor-Marsá, Maria V. Moneta, Albert Sanchez-Niubo, Stefanos Tyrovolas, Iago Giné-Vázquez, Josep M. Haro, Somnath Chatterji, Martin Bobak, Jose L. Ayuso-Mateos, Holger Arndt, Ilona Koupil, Jerome Bickenbach, Seppo Koskinen, Beata Tobiasz-Adamczyk, Demosthenes Panagiotakos, Alberto Raggi

**Affiliations:** 1 Neurology, Public Health and Disability Unit, Fondazione IRCCS Istituto Neurologico Carlo Besta, Via Celoria 11, 20133, Milan, Italy; 2 Parc Sanitari Sant Joan de Déu, Fundacion Sant Joan de Deu, Barcelona, Spain; 3 Instituto de Salud Carlos III, Centro de Investigación Biomédica en Red de Salud Mental, CIBERSAM, Madrid, Spain; 4 Department of Nutrition and Dietetics, School of Health Science and Education, Harokopio University, Athens, Greece; 5 Information, Evidence and Research, World Health Organization, Geneva, Switzerland; 6 Research Department of Epidemiology and Public Health, University College London, London, UK; 7 Department of Psychiatry, Universidad Autónoma de Madrid, Madrid, Spain; 8 Hospital Universitario de La Princesa, Instituto de Investigación Sanitaria Princesa, Madrid, Spain; 9 SPRING TECHNO GMBH & Co. KG, Bremen, Germany; 10 Department of Public Health Sciences, Centre for Health Equity Studies, Stockholm University, Stockholm, Sweden; 11 Department of Public Health Sciences, Karolinska Institutet, Stockholm, Sweden; 12 Department of Health Sciences and Health Policy, University of Lucerne, Lucerne, Switzerland; 13 Swiss Paraplegic Research, Nottwil, Switzerland; 14 Finnish Institute for Health and Welfare (THL), Helsinki, Finland; 15 Department of Epidemiology and Population Studies, Faculty of Health Sciences, Jagiellonian University Medical College, Krakow, Poland

**Keywords:** Pain, Projection, Bayesian age period cohort model, Headache disorders, Musculoskeletal disorders, Employment

## Abstract

**Background:**

Pain is a common symptom, often associated with neurological and musculoskeletal conditions, and experienced especially by females and by older people. The aims of this study are to evaluate the temporal variations of pain rates among general populations for the period 1991–2015 and to project 10-year pain rates.

**Methods:**

We used the harmonized dataset of ATHLOS project, which included 660,028 valid observations in the period 1990–2015 and we applied Bayesian age–period–cohort modeling to perform projections up to 2025. The harmonized Pain variable covers the content “self-reported pain experienced at the time of the interview”, with a dichotomous (yes or no) modality.

**Results:**

Pain rates were higher among females, older subjects, in recent periods, and among observations referred to cohorts of subjects born between the 20s and the 60s. The 10-year projections indicate a noteworthy increase in pain rates in both genders and particularly among subjects aged 66 or over, for whom a 10–20% increase in pain rate is foreseen; among females only, a 10–15% increase in pain rates is foreseen for those aged 36–50.

**Conclusions:**

Projected increase in pain rates will require specific interventions by health and welfare systems, as pain is responsible for limited quality of subjective well-being, reduced employment rates and hampered work performance. Worksite and lifestyle interventions will therefore be needed to limit the impact of projected higher pain rates.

## Introduction

Pain is defined as “a distressing experience associated with actual or potential tissue damage with sensory, emotional, cognitive, and social components” [1] and is one the most common symptoms. It can be associated with many conditions, including some highly prevalent and disabling ones, such as headaches, musculoskeletal diseases or injuries, but also to sequelae of other disorders, such as diabetes [2]. These conditions often have long-lasting duration: they begin in adulthood, endure for several years and often worsen, and are associated with disability [3–5]. Pain rates are expected to rise due to the increased prevalence of musculoskeletal and neurological diseases, increased life expectancy and increased proportion of elderly in societies [6].

The results of epidemiological studies show that the prevalence of pain-associated conditions is between 10% and 40% approximately [7–17]: the only exception to this is for the one-year prevalence of tension-type headache, which peaked up to 60% [13–15]. Literature also show that pain is more frequently experienced by females and by older people, which is consistent with the epidemiological presentation of the main drivers of pain, i.e. headache and musculoskeletal disorders. Based on these figures, it could be hypothesized that most of the general population would report pain as a daily or near-daily problem. However, reliable information on the presence and time trend of pain in the general population, irrespective of underlying health conditions, is limited to few studies. A recent one, which analyzed 18-year trends in the overall rates of noncancer pain prevalence in the U.S., showed that the proportion of adults reporting pain due to painful health conditions increased from 32.9% to 41.0% between 1997 and 98 and 2013–14 [18].

Understanding such a trend, as well as the projections towards future periods, is of relevance for the management of pain as a symptom, particularly for the risk of overuse of medications such as opioids [18–20]. In addition to this, issues connected to worse health outcome, e.g. higher disability, reduced employment rates and loss of productivity are also of relevance [21–28]. This is, in turn, an important determinant of economic burden, which is high because of both per-case cost and general prevalence of pain-related conditions [29–33]. However, while higher pain rates among females and among older subjects, and occasionally differences by period have been shown, with higher pain rates in recent periods [6, 18], information on pain rates by cohort is basically lacking. The integration of gender, age, period and cohort data is of core importance to produce projections on pain rates in the general population.

The aims of this study are to evaluate the temporal variations of pain rates among the general populations for the period 1991–2015 and to predict future rates up to 2025. These variations were analyzed by gender considering age, periods (year of survey) and cohorts (birth year), as derived by the ATHLOS project (Ageing Trajectories of Health – Longitudinal Opportunities and Synergies) harmonized dataset, which includes data collected in different studies carried out in the five continents.

## Materials and methods

### ATHLOS project and the harmonized dataset

ATHLOS project was funded by the European Union’s Horizon 2020 Research and Innovation Program, and it aims to achieve a better understanding of the impact of ageing on health. To achieve this result, a new cohort has been composed from harmonized datasets of existing international longitudinal cohorts related to health and ageing (see: https://github.com/athlosproject/athlos-project.github.io). The harmonized dataset includes records of participants from 17 different studies and is fully described elsewhere [34]: most of these studies were run between 2000 and 2010 and have at least two waves, but there are both older and more recent ones, as well as studies with one wave only. The harmonized ATHLOS mega-dataset comprises approximately 411,000 respondents. Most of the studies whose data are included in ATHLOS dataset were from high-income countries and upper-middle-income countries: the only exceptions are India and Ghana.

ATHLOS harmonized dataset is composed of a wide range of variables covering a variety of health conditions, sociodemographic variables, personal functioning and contextual factors, which are usually assessed in population studies. The ATHLOS dataset variables were classified in the following domains: sociodemographic and economic characteristics, lifestyle and health behaviors, health status and functional limitations, diseases, living status, physical measures, psychological measures, laboratory measures, social environment and life events, and administrative information [34].

Pain is included within health status and functional limitations and was measured in 14 out of the 17 studies, with different approaches (please refer to supplementary materials for information on the distribution of pain variable across studies and countries). Some studies, e.g. the Collaborative Research on Ageing in Europe [35] and the China Health and Retirement Longitudinal Study [36], addressed it in terms of pain severity (i.e. None, Mild, Moderate, Severe, Extreme or other similar formats). Other studies, e.g. the Australian Longitudinal Study of Aging [37] and the Survey of Health, Ageing and Retirement in Europe [38], dichotomously addressed the presence of pain (i.e. yes or no), sometimes addressing the idea that pain is “often experienced”, such as in the Irish Longitudinal Study of Ageing [39]. The harmonization procedure is aimed to generate inferentially equivalent content across studies so to make the content of variables collected in different studies uniform. In the case of pain variable, the content was “self-reported pain experienced at the time of the interview”, and the variable modality was dichotomous.

### Age period cohort analysis

Age period cohort (APC) models are commonly used to analyze and project rates [40, 41]. APC models account for these processes on three time scales: age, year of survey (period) and year of birth (cohort). The period and cohort effects are both surrogates for exposure to external factors. Period effects include environmental and diagnostic factors. For example, the introduction of a new diagnostic procedure may lead to a jump in disease incidence across all age groups. Cohort effects represent risk factors that change over time and may have a delayed effect on disease outcomes. For example, lifestyle factors, such as alcohol and tobacco consumption, can manifest themselves as cohort effects [41].

Data were organized by five-year periods from 1991 to 2015, five-year age-groups from the group 31–35 to the 96–100 one and stratified by gender. In a preliminary analysis, age-specific trend rates by gender were computed. Trend rates vary between 0 and 1, with higher values indicating higher proportion of pain reporting in specific subgroups and observations, i.e. among males and females, by age, cohort and periods. Later, APC models were fitted to analyze the joint effects of the age, period, and cohort, expressed in Rate Ratio (RR) terms. RR is a relative measure that allows to identify potential protective (if RR < 1) and risk factors (if RR > 1), in this case related to particular ages, periods or cohorts. Pain counts *y*_*ijk*_ in age group *i* at time point *j* in *k*^*th*^ cohort can be assumed to be Poisson distributed, i.e.,

$$ {\mathrm{y}}_{\mathrm{ijk}}=\mathrm{Po}\left({\mathrm{n}}_{\mathrm{ijk}}{\uplambda}_{\mathrm{ijk}}\right),\mathrm{where}\ \mathrm{i}=1,\dots, \mathrm{I};\mathrm{j}=1,\dots, \mathrm{J};\mathrm{k}=1,\dots, \mathrm{K}, $$

with mean *n*_*ijk*_*λ*_*ijk*_, where *n*_*ijk*_ denotes the corresponding subjects at risk. In our application, the age index *i* run from 1 to *I* = 14 (both in males and females), while the period index *j* run from 1 to *J = 5*. Concerning the cohort index *k*, it depended on the age group and period index, but also on their intervals width [42, 43] and it was defined as *M* × *(I* − *i)* + *j*, where *M* indicated the ratio between the width in years of the age group and the period intervals [44]. In our application *M* was equal to [5 years] / [5 years] = 1, and the cohort index *k*, following the previous formula where the age groups were fourteen and the periods are five, generated *K = 1 × (14–1) + 5 = 18* cohorts, i.e., the cohort index run from 1 to *K = 18*, but only 9 not overlapping (i.e., 1891–1900, 1901–1910,…,1971–1980). Given that, the model was specified as

$$ \mathrm{Log}\left({\uplambda}_{\mathrm{i}\mathrm{jk}}\right)={\upmu}_{\mathrm{i}\mathrm{jk}}=\upmu +{\upalpha}_{\mathrm{i}}+{\upbeta}_{\mathrm{j}}+{\upgamma}_{\mathrm{k}} $$

Here *μ* represented the general level (intercept), and *α*_*i*_, *β*_*j*_, *γ*_*k*_ denoted age, period, and cohort random effects (to be estimated), respectively [45].

A fully Bayesian approach based on Integrated Nested Laplace Approximations (INLA) was considered for model fitting and inference [46]. A Bayesian APC model provides a more robust methodology compared to a log-linear model, particularly for the prediction of future occurrence [47–49]. Indeed, in our case, Bayesian age–period–cohort modeling was also used to perform projections, and relative credible intervals (CI), of the pain symptom rates in the time interval 2015–2025, extrapolating the trend of rate from 1990 to 2015 and considering the subjects at risk relative to the last period 2011–2015. For each projection, different degrees of CI were reported, the closer to the predicted mean being 10% CI and the largest being 95% CI.

The model had some a priori assumptions: (i) log-RR for each effect summed to zero over the observed interval; (ii) the expected effects were hypothesized to be constant, so that both the large and the small deviations from a constant rate are detected. The Bayesian APC model also considered hyper-parameters of random walk type of first (RW1) and second order (RW2) [50] on which log-gamma prior distributions were elicited. We intentionally used highly non-informative log-gamma prior distributions (with parameters equal to 1 and 0.00005) in order to endorse and make more credibility to harmonized ATHLOS dataset and avoid the imposition of assumptions for which no a priori knowledge was available. A prior distribution on an overdispersion parameter was also elicited by an independent log-gamma with parameters equal to 1 and 0.005.

Parameter estimates in terms of mean and median were obtained. Finally, to select the best model among the different proposals, the Deviance Information Criterion (DIC, lower was better) [51] were computed. In particular, different specification as age period and age cohort models, and different combinations of priors of RW1 and RW2 [50] were probed by considering the random effects of age, period and cohort. In case RW1 and RW2 priors provided very similar DICs, we decided to select the complete model with RW2 priors because is a standard choice as natural target for smoothing [52, 53].

The technique was implemented in the software R v 3.5.2 [54] through the packages R-INLA (www.r-inla.org) [55–57] and Bayesian APC [50] for the projections.

## Results

In total 660,028 valid observations were used, of whom 293,484 reported some degree of pain, the rate being 44.5% (see Table 1): the rate of pain was higher among observations referred to females than to males. As shown in Table 2, the average age of participants at the time of observation was comprised between 64 and 65 years across all periods, with the exception of the 1991–1995 one, where the average age was approximately 5 years below that of the other periods. Supplementary Tables S1-S4 report distribution of all observations and of observations with pain by period and by 5-years age groups; supplementary Tables S5-S8 report the same information not aggregated by 5-years periods.

**Table 1:** Presence of pain in the general population included in ATHLOS harmonized dataset by age class

Age class		Males	Females	Total
**31–50**	Observations	18,287	37,510	55,797
	Cases with pain	6085	14,886	20,971
	% cases with pain	33.3%	39.7%	37.6%
**51–70**	Observations	182,094	223,289	405,383
	Cases with pain	70,425	106,939	177,364
	% cases with pain	38.7%	47.9%	43.8%
**> 70**	Observations	83,389	115,459	198,848
	Cases with pain	34,331	60,818	95,149
	% cases with pain	41.2%	52.7%	47.8%
**Total**	Observations	283,770	376,258	660,028
	Cases with pain	110,841	182,643	293,484
	% cases with pain	39.1%	48.5%	44.5%

**Table 2:** Age by period

Period	Males	Females	Total
**1991–1995**	60.6 ± 8.7	58.5 ± 10.1	59.4 ± 9.6
**1996–2000**	64.8 ± 11.0	64.8 ± 12.5	64.8 ± 11.9
**2001–2005**	64.6 ± 10.2	64.1 ± 11.5	64.3 ± 10.9
**2006–2010**	65.1 ± 10.9	65.0 ± 11.9	65.1 ± 11.5
**2011–2015**	64.9 ± 10. 7	64.8 ± 11.5	64.8 ± 11.2
**Total**	64. 7 ± 10.6	64.4 ± 11.7	64.5 ± 11.3

**Note.** Data are expressed in years as mean ± standard deviations

**Fig. 1:**
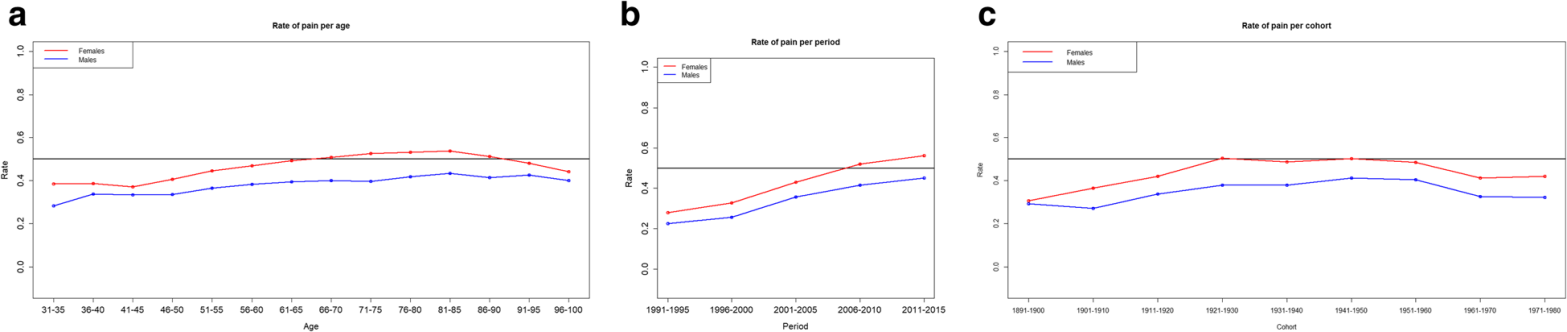
Observed rate of pain in males and females by age, period and cohort**. a**: pain rates by age. **b**: pain rates by period. **c**: pain rate by cohort. Note. The dark central line indicates rate = 0.5

Figure 1 reports the observed pain rates by age, period and cohort for males and females. Among males rates by age varied approximately between 0.3 and 0.4, increasing with age and remaining basically stable after 80 years of age; among females, rates by age varied approximately between 0.4 and 0.54, peaking at the age class 81–85 and declining thereafter. With regard to rates by periods, a consistent increase was observed in both genders: among males it varied between 0.2 and 0.4, among females between 0.3 and 0.5. Regarding rates by cohort, it has parabolic shapes in both genders: among males the peak was around 0.4 for the cohort born in 1941–1950, among females it was around 0.5 for the cohorts born between 1921 and 1950.

**Fig. 2:**
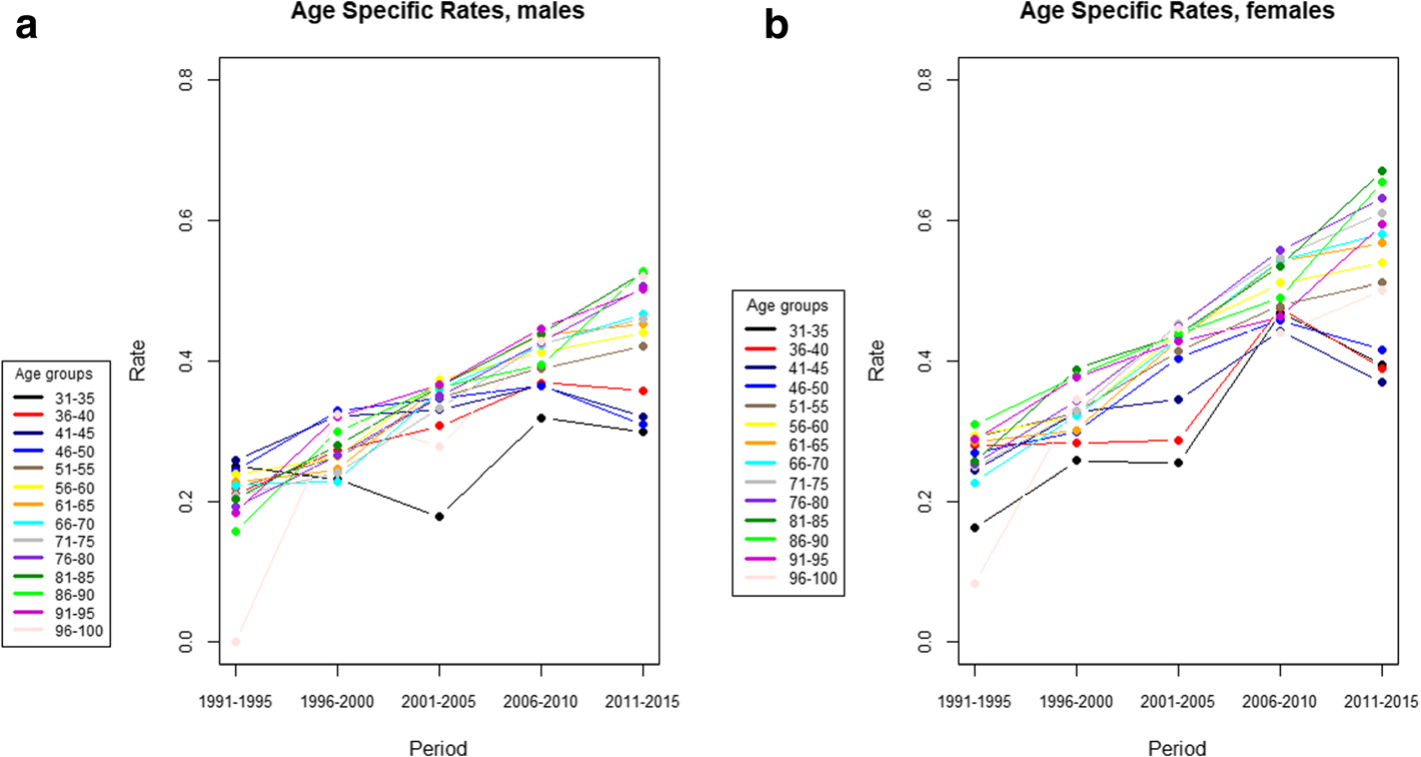
Observed pain rate in males and females in the ATHLOS dataset. **a**: age-specific pain rates by age for males. **b**: age-specific pain rates by age for females

Age-specific rates by gender and per period are shown in Fig. 2. Rates were higher among females and, in both genders, were higher among older subjects. A trend related to the period was observed, in both genders and across all age groups, with participants recruited in more recent periods experiencing pain with a higher frequency. An exception to this is observed for participants aged below 50 for whom, in the period 2010–2015, a decline in trend was observed in both genders.

**Fig. 3:**
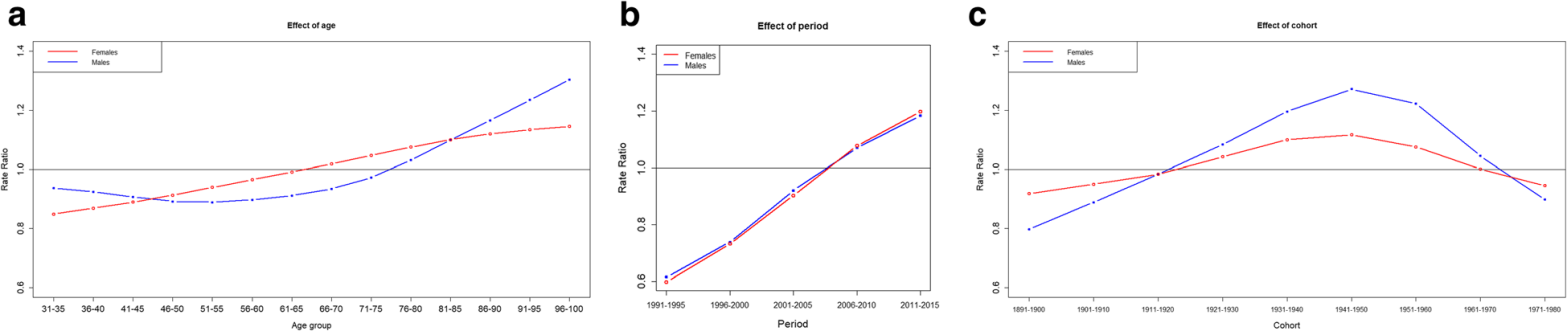
Estimated effects of age, period and cohort in rate ratio terms by gender**. a**: estimated effect of age. **b**: estimated effect of period. **c**: estimated effect of cohort. Note. The dark central line indicates RR = 1

Figure 3 shows the results of the APC models. The effect of age was different among males and females: in fact, while a consistent increasing trend was observed in females, with age becoming a risk factor for pain in the seventh decade of life, the age effect in males decreased from the first age group up to the 50–55 group and then it increased, becoming a risk factor in the eighth decade of life. The period effect was constantly growing in both males and females and it became a risk factor for pain from the period 2006–2010 onwards. The cohort effect had a parabolic shape: being born between the 20s and the 60s was a risk factor for pain in both genders. The selected models for both males (DIC = 650.6) and females (DIC = 723.8) involved the complete age period cohort models and the choice of RW2 priors (see supplementary Table S9). It is worth to point out that the estimated decreasing trend for the youngest age groups in males could be due to the small number of periods in relation to the age groups. In order to account for that, we have also performed a sensitivity analysis where the periods were non-aggregated (data are included in supplemental materials, Tables S5-S8). The results of this last analysis were very similar to that of those carried out with aggregated data: only for the period effects differences on the trends that presented a more irregular increase were detected (see Fig. S1). Moreover, because of data sparsity, sensitivity analysis could not produce projection as estimates intervals would have been too wide and unstable.

**Fig. 4:**
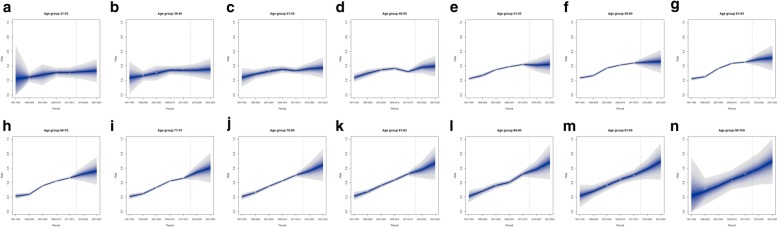
Pain rates and 10-years pain rate projection per age group in males**. a**: pain rate projection, males, age 31–35. **b**: pain rate projection, males, age 36–40. **c**: pain rate projection, males, age 41–45. **d**: pain rate projection, males, age 46–50. **e**: pain rate projection, males, age 51–55. **f**: pain rate projection, males, age 56–60. **g**: pain rate projection, males, age 61–65. **h**: pain rate projection, males, age 66–70. **i**: pain rate projection, males, age 71–75. **j**: pain rate projection, males, age 76–80. **k**: pain rate projection, males, age 81–85. **l**: pain rate projection, males, age 86–90. **m**: pain rate projection, males, age 91–95. **n**: pain rate projection, males, age 96–100. Note. The predictive mean is shown as solid line. The different shadings indicate pointwise credible intervals. The central interval represents 10% CI, and the largest interval 95% CI. Observed rates are shown as a filled circle. The vertical dashed line indicates where prediction started

**Fig. 5:**
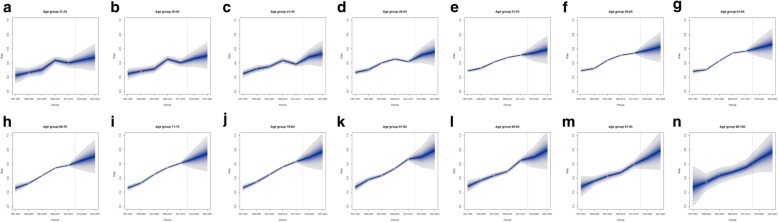
Pain rates and 10-years pain rate projection per age group in females**. a**: pain rate projection, females, age 31–35. **b**: pain rate projection, females, age 36–40. **c**: pain rate projection, females, age 41–45. **d**: pain rate projection, females, age 46–50. **e**: pain rate projection, females, age 51–55. **f**: pain rate projection, females, age 56–60. **g**: pain rate projection, females, age 61–65. **h**: pain rate projection, females, age 66–70. **i**: pain rate projection, females, age 71–75. **j**: pain rate projection, females, age 76–80. **k**: pain rate projection, females, age 81–85. **l**: pain rate projection, females, age 86–90. **m**: pain rate projection, females, age 91–95. **n**: pain rate projection, females, age 96–100. Note. The predictive mean is shown as solid line. The different shadings indicate pointwise credible intervals. The central interval represents 10% CI, and the largest interval 95% CI. Observed rates are shown as a filled circle. The vertical dashed line indicates where prediction started

Figures 4 and 5 show observed and predicted rates for all age groups and for males and females, respectively. For both males and females, the projection is indicative of an increase in pain rates between 2015 and 2025, and such an increase was wider among older subjects. Among males, the projected increase over the decade was higher than 10% in all age groups from the 66–70 one, and get close to 20% for the age groups 91–95 and 96–100 (Fig. 4m-n). The same applies to females, for whom however the projection over the decade was higher than 20% in the oldest group (Fig. 5n). Moreover, an increase comprised between 10% and 15% over the decade was also observed among females for the age groups comprised between 36 and 50 years (Fig. 5b-d).

## Discussion

The results of this study confirm part of the finding of previous literature, namely higher rates of pain among females and among older subjects, and reinforce the few available data on period analysis by showing higher pain rates in more recent periods, with specific reference to the observations referred to older subjects. In addition to this, our results show that pain rates were higher among the observations referred to cohorts of subjects born between the 20s and the 60s (i.e. between 1921 and 1970). Finally, the most innovative aspect of our work lies in the 10-year projection for pain rates, which indicate a noteworthy increase in pain rates across all age groups and in both genders. Among males aged 66 and over, the increase is projected to be by 10–20% over the decade, and the same is for females: in addition to this, however, also females aged 36–50 will likely face a 10–15% increase in pain rates.

Taken as a whole, our results tell the story of an expansion of pain-related morbidity, irrespective of its aetiology. Chronic pain prevalence has been occasionally addressed, but available reports show that it is increasing with age and through the years as an effect of population ageing [58, 59]. In addition to the known effect of age, some reports have also shown that people enrolled in recent years in population studies tend to report higher pain rates, i.e. an effect of age [6, 18] which may underlie differences in pain reporting, that might be due to cultural or biological issues. Our data do not enable to make hypotheses on what is causing such a period effect, but it has to be acknowledged that the prevalence of some conditions is rising worldwide. Examples of this include diabetic neuropathy, which affects up to 50% of patients with diabetes [60] and is expected to increase in consideration of the rising prevalence of diabetes worldwide [61, 62], and musculoskeletal conditions, which are among the main pain drivers, and represent approximately 19% of non-communicable diseases in terms of prevalence and 20% in terms of disability [2].

In consideration of the higher impact of such conditions among older subjects, and in consideration of global population ageing, actions aimed to prevent and control pain are and will be more and more needed. It is interesting to notice the increase in pain rates which is specific for females aged 36–50. This age group is at higher risk of having migraine or tension-type headache [3, 63–66]. It has to be noted that, as shown by GBD data [3], the decrease in prevalence of headache disorders begins after the age of 30, but the decline is particularly pronounced after the age of 50, particularly among women; this may be the reason for the differential increase by gender in the 36–50 age group. In addition to this, there are biological differences that make women to be exposed to specific pain, such as cyclic menstrual pain or menstrual migraine, which affects 22% to 62% of female migraineurs [67, 68]. Such an increase in pain rates will also be accompanied by an increase in the amount of the portion of population in that age group (third to fifth decade of life) in high and upper-middle income countries. In fact, in high-income countries, the median age between 1990 and 2015, moved from 33.4 to 40.4, and is projected to span between 41.5 and 43.5 in 2020–2030; in upper-middle income countries, the median age between 1990 and 2015, moved from 24.5 to 33.9, and is projected to span between 35.6 and 39.9 in 2020–2030 [69]. Therefore, it can be hypothesized that headache disorders will contribute more and more to the overall burden of pain in general populations. In addition to this, headache disorders and back pain, which are among the most prevalent and disabling conditions [2], show considerable rates of comorbidity, with odds of association comprised between 1.55 and 8.00 [70].

It is reasonable to hypothesize that in the older age groups, the difference in the increase of pain might be associated to the higher impact of some skeletal conditions, such as osteoporosis, and to the associated higher fracture rates. In fact, osteoporosis is approximately three-fold more prevalent in women than in men [71], and women older than 60 years have a 44% lifetime risk of fractures, compared to 25% among men of the same age [72].

Increasing pain rates will most likely be associated with increased healthcare utilization and consumption of different kinds of analgesics [73–75], and in particularly opioids, which however may in turn produce negative health outcomes and a further increase of healthcare use [76, 77]. In addition to this, pain is associated with reduced work productivity and sick leave [22, 26, 27, 78–81], early retirement and reduced employment rates [21, 25, 28, 82, 83]. The old-age dependency ratio, i.e. the ratio of the population aged 65 years or over to the population aged 15–64 -- referred to as the number of dependants per 100 persons of working age -- has been constantly increasing and is projected to further increase in both high-income and upper-middle-income countries. In high-income countries the dependency ratio increased from 18.3 to 25.7 between 1990 and 2015, and is projected to span between 25.8 and 36.3 between 2020 and 2030; in upper-middle-income countries the increase was from 8.1 to 10.5 between 1990 and 2015, and the rate will span between 12.3 and 16.2 between 2020 and 2030 [69]. Thus, our projections are not only indicative of an increased risk of future worse health status, but also of higher healthcare expenditure and overall financial burden which the health and welfare systems of high and upper-middle income countries, i.e. countries from which most of observations included in ATHLOS dataset were drawn [34], likely will struggle to bear.

Interventions are therefore needed to prevent pain and limit the impact of projected higher pain rates, which should include worksite interventions [84–89], lifestyle interventions and control over prescribed drugs [89–92]. Worksite intervention should be aimed to limit work cessation as well as enhance work productivity, and should ideally to be tailored to the features of each individual. Practically, these interventions can be tailored to the features of specific disorders as well, and may thus involve specific actions. A synthesis of the most effective strategies included among available literature [84–92] is beyond the aims of this study, but some general considerations can be made. Evidence exist that effective interventions act upon different levels, i.e. both patients and the workplaces, and involve several stakeholders, including treating pain specialists, occupational physicians and employers, and the most commonly reported strategies include appropriate pharmacotherapy, provision of ergonomic furniture, patients’ education on pain management, relaxation/posture exercises. Lifestyle interventions are aimed to reduce factors contributing to pain exacerbation and pain triggers, and most of them target diet and physical activity, as well as appropriate drug prescription and adherence to treatment. Finally, preliminary research evidence is available on the effectiveness of behavioral treatments, alone or as add-on to medical ones, for chronic pain control [93–95]. More research is needed in this field, that seems however promising and could provide a substantial contribution to control pain experience and reduce the consumption of medications.

Some limitations have to be acknowledged. The age period cohort models are commonly used to analyse and project mortality or morbidity rates from health registers to routinely collect demographic rates. However, the data herein reported do not derive from registries but from cross-sectional and longitudinal surveys, harmonized in the ATHLOS dataset. This entails that the population is not the same across periods, and therefore the sample sizes were different. Moreover, our analysis did not consider population dynamics either. In addition to this, it has to be remembered that our analysis is based on a harmonized dataset, whose core definition of pain variable is “self-reported pain experienced at the time of the interview”, with a dichotomous output. This creates an important limitation, namely the fact that this variable does not account for two issues of relevance in pain experience. The first is the severity and impact of pain, which spans between mild and disabling; the second is the frequency with which pain is experienced, which might be daily or near-daily, such as pain due to musculoskeletal conditions, episodic with variable frequency, such as in the case of headache disorders, or occasional. Finally, we interpreted the results of pain trends in light of the trends that can be reasonably expected for musculoskeletal and headache disorders in reason of their prevalence in the general population. However, none of the original pain-related variables included specification of pain in terms of aetiology or location.

## Conclusion

In conclusion, we reported data on the temporal variations of pain rates among the general population for the period 1991–2015 and predicted 10-years future rates. Our results are based on a very large international dataset, which included data from populations from the five continents. Results show that pain rates were higher among females and among older subjects, and that rates were also higher among the respondents enrolled in more recent periods. Finally we showed that the trends of pain are increasing, in particular among females and among older subjects, for whom a 10–20% increase is projected over the 10-year period.

Pain is strongly associated with reduced employment rates and hampered work performance which, in consideration of population ageing and projected increase of dependency ratio of older people on people of working age, will require specific actions by health and welfare systems. Worksite and lifestyle interventions will be needed to limit the impact of projected higher pain rates.

## Supplementary information

**Additional file 1. **Trends Pain – Supplementary Materials – February 10, 2020**.** supplementary results not included in the full text.

## Data Availability

The datasets generated and/or analysed during the current study are not publicly available due restrictions imposed by part of the owners (COURAGE in Europe, HAPIEE, ALSA, the Health 2000 and 2011 Surveys- Finland, ENRICA and the 10/66 study), but may be available from the corresponding author upon reasonable request and once consent form ATHLOS project intellectual property and dissemination board is obtained.

## Abbreviations

AP: Age Period Cohort
ATHLOS: Ageing Trajectories of Health – Longitudinal Opportunities and Synergies
CI: Credible Intervals
DIC: Deviance Information Criterion
INLA: Integrated Nested Laplace Approximations
RR: Rate Ratio
RW1: Random Walk type of first order
RW2: Random Walk type of second order
