# Supplementary material for: Pain rates in general population for the period 1991–2015 and 10-years prediction: results from a multi-continent age-period-cohort analysis
Source: J Headache Pain. 2020 May 13;21(1):52. doi: 10.1186/s10194-020-01108-3 (PMC7218619; doi:10.1186/s10194-020-01108-3)
Supplement: Supplementary file 1 — Additional file 1. Trends Pain – Supplementary Materials – February 10, 2020. supplementary results not included in the full text. [file 10194_2020_1108_MOESM1_ESM.docx]

**Trends Pain – Supplementary Materials – February 10, 2020**

**Authors**: Guido D, Raggi A, Mellor B, Moneta MV, Sanchez-Niubo A, Tyrovolas S, Giné-Vázquez I, Haro JM, Chatterji S, Bobak M, Prince M, Ayuso-Mateos JL, Arndt H, Scherbov S, Koupil I, Bickenbach J, Koskinen S, Tobiasz-Adamczyk B, Gheno I, Panagiotakos D, Leonardi M. **Manuscript Title:** Pain rates in general population for the period 1991-2015 and 10-years prediction: result from a multi-continent age-period-cohort analysis. **Journal:** The Journal of Headache and Pain

**Table S1 – Presence of pain symptom for males aged 31-100 for five-year age group, period 1991-2015 in the ATHLOS dataset**

| \| **Age groups** \| **31-35** \| **36-40** \| **41-45** \| **46-50** \| **51-55** \| **56-60** \| **61-65** \| **66-70** \| **71-75** \| **76-80** \| **81-85** \| **86-90** \| **91-95** \| **96-100** \| **Total** \| \| --- \| --- \| --- \| --- \| --- \| --- \| --- \| --- \| --- \| --- \| --- \| --- \| --- \| --- \| --- \| --- \| \| **Periods** \|  \|  \|  \|  \|  \|  \|  \|  \|  \|  \|  \|  \|  \|  \|  \| \| **1991-1995** \| 1 \| 4 \| 15 \| 81 \| 800 \| 915 \| 516 \| 158 \| 175 \| 118 \| 65 \| 21 \| 7 \| 0 \| **2876** \| \| **1996-2000** \| 70 \| 86 \| 116 \| 197 \| 700 \| 1385 \| 1263 \| 798 \| 692 \| 580 \| 385 \| 215 \| 59 \| 11 \| **6557** \| \| **2001-2005** \| 10 \| 39 \| 127 \| 1099 \| 3457 \| 3874 \| 3984 \| 3877 \| 2372 \| 1802 \| 1082 \| 462 \| 140 \| 18 \| **22343** \| \| **2006-2010** \| 140 \| 221 \| 342 \| 1533 \| 5150 \| 6304 \| 5894 \| 6048 \| 5430 \| 3773 \| 2160 \| 869 \| 281 \| 50 \| **38195** \| \| **2011-2015** \| 110 \| 155 \| 365 \| 1374 \| 5036 \| 6790 \| 7140 \| 6336 \| 5303 \| 4243 \| 2659 \| 1069 \| 252 \| 38 \| **40870** \| \| **Total** \| **331** \| **505** \| **965** \| **4284** \| **15143** \| **19268** \| **18797** \| **17217** \| **13972** \| **10516** \| **6351** \| **2636** \| **739** \| **117** \| **110841** \| |  |  |  |  |  |  |  |  |  |  |  |  |  |  |  |  |  |  |  |  |  |
| --- | --- | --- | --- | --- | --- | --- | --- | --- | --- | --- | --- | --- | --- | --- | --- | --- | --- | --- | --- | --- | --- | --- | --- | --- | --- | --- | --- | --- | --- | --- | --- | --- | --- | --- | --- | --- | --- | --- | --- | --- | --- | --- | --- | --- | --- | --- | --- | --- | --- | --- | --- | --- | --- | --- | --- | --- | --- | --- | --- | --- | --- | --- | --- | --- | --- | --- | --- | --- | --- | --- | --- | --- | --- | --- | --- | --- | --- | --- | --- | --- | --- | --- | --- | --- | --- | --- | --- | --- | --- | --- | --- | --- | --- | --- | --- | --- | --- | --- | --- | --- | --- | --- | --- | --- | --- | --- | --- | --- | --- | --- | --- | --- | --- | --- | --- | --- | --- | --- | --- | --- | --- | --- | --- | --- | --- | --- | --- | --- | --- | --- | --- | --- | --- | --- | --- | --- | --- | --- | --- | --- | --- | --- | --- | --- | --- | --- | --- | --- | --- |

**Table S2 – Male population aged 31-100 for five-year age group, period 1991-2015 in the ATHLOS dataset**

| **Age groups** | **31-35** | **36-40** | **41-45** | **46-50** | **51-55** | **56-60** | **61-65** | **66-70** | **71-75** | **76-80** | **81-85** | **86-90** | **91-95** | **96-100** | **Total** |
| --- | --- | --- | --- | --- | --- | --- | --- | --- | --- | --- | --- | --- | --- | --- | --- |
| **Periods** |  |  |  |  |  |  |  |  |  |  |  |  |  |  |  |
| **1991-1995** | 4 | 19 | 58 | 332 | 3645 | 3844 | 2271 | 708 | 843 | 613 | 320 | 134 | 38 | 3 | **12832** |
| **1996-2000** | 303 | 316 | 361 | 599 | 2665 | 5270 | 5157 | 3498 | 2889 | 2188 | 1377 | 718 | 184 | 34 | **25559** |
| **2001-2005** | 56 | 127 | 384 | 3168 | 9939 | 10399 | 10885 | 10810 | 7126 | 5150 | 2975 | 1271 | 383 | 65 | **62738** |
| **2006-2010** | 440 | 600 | 940 | 4198 | 13210 | 15282 | 13480 | 14314 | 12795 | 8842 | 4923 | 2207 | 629 | 117 | **91977** |
| **2011-2015** | 368 | 433 | 1141 | 4440 | 11965 | 15429 | 15756 | 13567 | 11515 | 8388 | 5061 | 2026 | 502 | 73 | **90664** |
| **Total** | **1171** | **1495** | **2884** | **12737** | **41424** | **50224** | **47549** | **42897** | **35168** | **25181** | **14656** | **6356** | **1736** | **292** | **283770** |

|  |  |  |  |  |  |  |  |  |  |  |  |  |  |  |
| --- | --- | --- | --- | --- | --- | --- | --- | --- | --- | --- | --- | --- | --- | --- |

**Table S3 – Presence of pain symptom for females aged 31-100 for five-year age group, period 1991-2015 in the ATHLOS dataset**

| **Age groups** | **31-35** | **36-40** | **41-45** | **46-50** | **51-55** | **56-60** | **61-65** | **66-70** | **71-75** | **76-80** | **81-85** | **86-90** | **91-95** | **96-100** | **Total** |
| --- | --- | --- | --- | --- | --- | --- | --- | --- | --- | --- | --- | --- | --- | --- | --- |
| **Periods** |  |  |  |  |  |  |  |  |  |  |  |  |  |  |  |
| **1991-1995** | 11 | 50 | 131 | 450 | 1305 | 1281 | 456 | 90 | 237 | 200 | 99 | 61 | 15 | 1 | **4387** |
| **1996-2000** | 99 | 145 | 252 | 534 | 1452 | 2075 | 1806 | 1166 | 1133 | 1087 | 972 | 534 | 192 | 39 | **11486** |
| **2001-2005** | 53 | 149 | 550 | 2589 | 5256 | 5610 | 5556 | 5460 | 3964 | 3003 | 2049 | 1005 | 343 | 82 | **35669** |
| **2006-2010** | 480 | 659 | 894 | 3437 | 8200 | 9381 | 9103 | 9655 | 8761 | 6708 | 3865 | 1926 | 635 | 139 | **63843** |
| **2011-2015** | 224 | 322 | 866 | 2991 | 8386 | 10482 | 10875 | 9344 | 8800 | 6955 | 4942 | 2270 | 676 | 125 | **67258** |
| **Total** | **867** | **1325** | **2693** | **10001** | **24599** | **28829** | **27796** | **25715** | **22895** | **17953** | **11927** | **5796** | **1861** | **386** | **182643** |

**Table S4 – Female population aged 31-100 for five-year age group, period 1991-2015 in the ATHLOS dataset**

| **Age groups** | **31-35** | **36-40** | **41-45** | **46-50** | **51-55** | **56-60** | **61-65** | **66-70** | **71-75** | **76-80** | **81-85** | **86-90** | **91-95** | **96-100** | **Total** |
| --- | --- | --- | --- | --- | --- | --- | --- | --- | --- | --- | --- | --- | --- | --- | --- |
| **Periods** |  |  |  |  |  |  |  |  |  |  |  |  |  |  |  |
| **1991-1995** | 68 | 179 | 537 | 1680 | 4467 | 4354 | 1602 | 397 | 953 | 791 | 387 | 197 | 52 | 12 | **15676** |
| **1996-2000** | 385 | 512 | 773 | 1785 | 4449 | 6442 | 5995 | 3621 | 3445 | 3172 | 2504 | 1410 | 510 | 113 | **35116** |
| **2001-2005** | 208 | 519 | 1596 | 6409 | 12669 | 12714 | 12893 | 12594 | 8758 | 6679 | 4685 | 2291 | 801 | 183 | **82999** |
| **2006-2010** | 1023 | 1385 | 2019 | 7502 | 17130 | 18367 | 16780 | 17767 | 16001 | 12045 | 7229 | 3927 | 1371 | 316 | **122862** |
| **2011-2015** | 568 | 827 | 2343 | 7192 | 16371 | 19446 | 19139 | 16092 | 14404 | 10999 | 7373 | 3463 | 1138 | 250 | **119605** |
| **Total** | **2252** | **3422** | **7268** | **24568** | **55086** | **61323** | **56409** | **50471** | **43561** | **33686** | **22178** | **11288** | **3872** | **874** | **376258** |

**Table S5 – Presence of pain symptom for males aged 31-100 for five-year age group, period 1992-2015 (non-aggregated years) in the ATHLOS dataset**

| **Age group** | **31-35** | **36-40** | **41-45** | **46-50** | **51-55** | **56-60** | **61-65** | **66-70** | **71-75** | **76-80** | **81-85** | **86-90** | **91-95** | **96-100** | **Total** |
| --- | --- | --- | --- | --- | --- | --- | --- | --- | --- | --- | --- | --- | --- | --- | --- |
| **Period** |  |  |  |  |  |  |  |  |  |  |  |  |  |  |  |
| **1992** | 0 | 2 | 11 | 56 | 408 | 401 | 194 | 54 | 26 | 16 | 13 | 5 | 1 | 0 | **1187** |
| **1993** | 1 | 0 | 0 | 3 | 51 | 50 | 32 | 4 | 8 | 4 | 3 | 1 | 0 | 0 | **157** |
| **1994** | 0 | 2 | 4 | 22 | 341 | 464 | 289 | 92 | 17 | 6 | 1 | 0 | 0 | 0 | **1238** |
| **1995** | 0 | 0 | 0 | 0 | 0 | 0 | 1 | 8 | 124 | 92 | 48 | 15 | 6 | 0 | **294** |
| **1996** | 1 | 1 | 3 | 15 | 146 | 394 | 328 | 127 | 92 | 77 | 87 | 46 | 13 | 2 | **1332** |
| **1997** | 0 | 0 | 0 | 1 | 6 | 14 | 6 | 3 | 0 | 0 | 0 | 0 | 0 | 0 | **30** |
| **1998** | 0 | 4 | 8 | 35 | 231 | 450 | 376 | 271 | 266 | 226 | 117 | 64 | 23 | 5 | **2076** |
| **1999** | 0 | 0 | 0 | 1 | 15 | 13 | 13 | 13 | 10 | 3 | 3 | 0 | 0 | 0 | **71** |
| **2000** | 69 | 81 | 105 | 145 | 302 | 514 | 540 | 384 | 324 | 274 | 178 | 105 | 23 | 4 | **3048** |
| **2001** | 7 | 24 | 37 | 104 | 464 | 396 | 326 | 318 | 209 | 158 | 77 | 51 | 13 | 3 | **2187** |
| **2002** | 3 | 5 | 21 | 76 | 419 | 598 | 791 | 653 | 453 | 369 | 236 | 126 | 26 | 2 | **3778** |
| **2003** | 0 | 1 | 38 | 434 | 709 | 880 | 818 | 692 | 216 | 182 | 100 | 38 | 10 | 2 | **4120** |
| **2004** | 0 | 8 | 26 | 444 | 1478 | 1582 | 1693 | 1745 | 1058 | 803 | 482 | 183 | 67 | 8 | **9577** |
| **2005** | 0 | 1 | 5 | 41 | 387 | 418 | 356 | 469 | 436 | 290 | 187 | 64 | 24 | 3 | **2681** |
| **2006** | 2 | 4 | 61 | 400 | 1018 | 1284 | 1277 | 1560 | 1313 | 881 | 545 | 222 | 72 | 9 | **8648** |
| **2007** | 124 | 193 | 223 | 682 | 2558 | 2751 | 2337 | 2138 | 1762 | 1086 | 622 | 229 | 80 | 18 | **14803** |
| **2008** | 4 | 10 | 15 | 216 | 535 | 891 | 870 | 1051 | 878 | 612 | 351 | 136 | 46 | 6 | **5621** |
| **2009** | 6 | 10 | 19 | 29 | 164 | 282 | 276 | 290 | 351 | 296 | 185 | 85 | 21 | 5 | **2019** |
| **2010** | 4 | 4 | 24 | 206 | 875 | 1096 | 1134 | 1009 | 1126 | 898 | 457 | 197 | 62 | 12 | **7104** |
| **2011** | 80 | 109 | 246 | 747 | 2712 | 3291 | 3274 | 2630 | 2202 | 1712 | 1092 | 438 | 90 | 19 | **18642** |
| **2012** | 8 | 17 | 27 | 104 | 897 | 1313 | 1548 | 1565 | 1360 | 1125 | 628 | 264 | 70 | 12 | **8938** |
| **2013** | 0 | 3 | 68 | 466 | 1270 | 1829 | 1900 | 1609 | 1353 | 1077 | 748 | 277 | 65 | 3 | **10668** |
| **2014** | 1 | 2 | 3 | 21 | 84 | 165 | 229 | 283 | 198 | 185 | 98 | 40 | 11 | 2 | **1322** |
| **2015** | 21 | 24 | 21 | 36 | 73 | 192 | 189 | 249 | 190 | 144 | 93 | 50 | 16 | 2 | **1300** |
| **Total** | **331** | **505** | **965** | **4284** | **15143** | **19268** | **18797** | **17217** | **13972** | **10516** | **6351** | **2636** | **739** | **117** | **110841** |

**Table S6 – Male population aged 31-100 for five-year age group, period 1992-2015 (non-aggregated years) in the ATHLOS dataset**

| **Age group** | **31-35** | **36-40** | **41-45** | **46-50** | **51-55** | **56-60** | **61-65** | **66-70** | **71-75** | **76-80** | **81-85** | **86-90** | **91-95** | **96-100** | **Total** |
| --- | --- | --- | --- | --- | --- | --- | --- | --- | --- | --- | --- | --- | --- | --- | --- |
| **Period** |  |  |  |  |  |  |  |  |  |  |  |  |  |  |  |
| **1992** | 1 | 10 | 39 | 234 | 1927 | 1777 | 846 | 251 | 163 | 113 | 107 | 63 | 11 | 2 | **5544** |
| **1993** | 1 | 0 | 3 | 17 | 268 | 230 | 130 | 25 | 39 | 35 | 31 | 19 | 2 | 0 | **800** |
| **1994** | 2 | 9 | 16 | 81 | 1450 | 1836 | 1293 | 400 | 73 | 24 | 5 | 0 | 0 | 0 | **5189** |
| **1995** | 0 | 0 | 0 | 0 | 0 | 1 | 2 | 32 | 568 | 441 | 177 | 52 | 25 | 1 | **1299** |
| **1996** | 2 | 6 | 12 | 52 | 628 | 1755 | 1577 | 614 | 450 | 351 | 322 | 176 | 35 | 9 | **5989** |
| **1997** | 0 | 0 | 1 | 3 | 17 | 63 | 54 | 18 | 7 | 0 | 0 | 0 | 0 | 0 | **163** |
| **1998** | 7 | 13 | 36 | 161 | 923 | 1776 | 1593 | 1288 | 1156 | 849 | 484 | 236 | 74 | 11 | **8607** |
| **1999** | 0 | 0 | 3 | 3 | 73 | 54 | 54 | 53 | 53 | 25 | 7 | 1 | 0 | 0 | **326** |
| **2000** | 294 | 297 | 309 | 380 | 1024 | 1622 | 1879 | 1525 | 1223 | 963 | 564 | 305 | 75 | 14 | **10474** |
| **2001** | 43 | 73 | 112 | 369 | 1513 | 1242 | 946 | 810 | 571 | 405 | 182 | 96 | 20 | 6 | **6388** |
| **2002** | 4 | 18 | 63 | 321 | 1392 | 1887 | 2506 | 2209 | 1639 | 1248 | 774 | 370 | 84 | 17 | **12532** |
| **2003** | 0 | 3 | 116 | 1146 | 2093 | 2265 | 2107 | 1764 | 685 | 474 | 240 | 110 | 30 | 7 | **11040** |
| **2004** | 9 | 31 | 78 | 1203 | 3934 | 3918 | 4345 | 4650 | 3099 | 2278 | 1307 | 503 | 177 | 25 | **25557** |
| **2005** | 0 | 2 | 15 | 129 | 1007 | 1087 | 981 | 1377 | 1132 | 745 | 472 | 192 | 72 | 10 | **7221** |
| **2006** | 9 | 14 | 208 | 1177 | 2828 | 3251 | 3107 | 3926 | 3182 | 2099 | 1279 | 573 | 151 | 28 | **21832** |
| **2007** | 373 | 506 | 535 | 1561 | 5621 | 5711 | 4604 | 4113 | 3146 | 2014 | 1071 | 379 | 119 | 21 | **29774** |
| **2008** | 24 | 33 | 67 | 697 | 1624 | 2454 | 2218 | 2759 | 2337 | 1570 | 946 | 472 | 135 | 19 | **15355** |
| **2009** | 23 | 30 | 50 | 88 | 424 | 742 | 651 | 768 | 1154 | 853 | 571 | 247 | 58 | 15 | **5674** |
| **2010** | 11 | 17 | 80 | 675 | 2713 | 3124 | 2900 | 2748 | 2976 | 2306 | 1056 | 536 | 166 | 34 | **19342** |
| **2011** | 258 | 281 | 753 | 2457 | 6476 | 7516 | 7159 | 5552 | 4688 | 3287 | 1969 | 768 | 153 | 26 | **41343** |
| **2012** | 43 | 67 | 100 | 315 | 2773 | 3729 | 4369 | 4177 | 3770 | 2815 | 1675 | 678 | 199 | 32 | **24742** |
| **2013** | 1 | 8 | 216 | 1536 | 2263 | 3298 | 3198 | 2500 | 2039 | 1523 | 964 | 383 | 94 | 7 | **18030** |
| **2014** | 3 | 8 | 12 | 55 | 250 | 463 | 609 | 750 | 544 | 470 | 264 | 108 | 28 | 6 | **3570** |
| **2015** | 63 | 69 | 60 | 77 | 203 | 423 | 421 | 588 | 474 | 293 | 189 | 89 | 28 | 2 | **2979** |
| **Total** | **1171** | **1495** | **2884** | **12737** | **41424** | **50224** | **47549** | **42897** | **35168** | **25181** | **14656** | **6356** | **1736** | **292** | **283770** |

**Table S7 – Presence of pain symptom for females aged 31-100 for five-year age group, period 1992-2015 (non-aggregated years) in the ATHLOS dataset**

| **Age group** | **31-35** | **36-40** | **41-45** | **46-50** | **51-55** | **56-60** | **61-65** | **66-70** | **71-75** | **76-80** | **81-85** | **86-90** | **91-95** | **96-100** | **Total** |
| --- | --- | --- | --- | --- | --- | --- | --- | --- | --- | --- | --- | --- | --- | --- | --- |
| **Period** |  |  |  |  |  |  |  |  |  |  |  |  |  |  |  |
| **1992** | 6 | 27 | 70 | 268 | 616 | 576 | 89 | 13 | 18 | 13 | 9 | 3 | 1 | 0 | **1709** |
| **1993** | 1 | 3 | 12 | 17 | 68 | 65 | 16 | 5 | 8 | 3 | 1 | 0 | 0 | 0 | **199** |
| **1994** | 4 | 20 | 49 | 163 | 619 | 638 | 342 | 9 | 3 | 0 | 0 | 0 | 0 | 0 | **1847** |
| **1995** | 0 | 0 | 0 | 2 | 2 | 2 | 9 | 63 | 208 | 184 | 89 | 58 | 14 | 1 | **632** |
| **1996** | 2 | 11 | 32 | 100 | 388 | 599 | 477 | 64 | 144 | 151 | 207 | 112 | 39 | 11 | **2337** |
| **1997** | 0 | 0 | 1 | 0 | 14 | 19 | 13 | 3 | 0 | 0 | 0 | 0 | 0 | 0 | **50** |
| **1998** | 3 | 20 | 48 | 148 | 486 | 643 | 556 | 434 | 418 | 377 | 304 | 173 | 57 | 16 | **3683** |
| **1999** | 0 | 0 | 3 | 8 | 24 | 23 | 17 | 14 | 14 | 13 | 7 | 3 | 1 | 0 | **127** |
| **2000** | 94 | 114 | 168 | 278 | 540 | 791 | 743 | 651 | 557 | 546 | 454 | 246 | 95 | 12 | **5289** |
| **2001** | 30 | 66 | 201 | 425 | 851 | 726 | 569 | 480 | 311 | 237 | 125 | 63 | 20 | 8 | **4112** |
| **2002** | 2 | 16 | 63 | 230 | 692 | 909 | 1014 | 911 | 726 | 612 | 463 | 262 | 80 | 16 | **5996** |
| **2003** | 15 | 27 | 128 | 823 | 1202 | 1203 | 1084 | 871 | 389 | 278 | 161 | 63 | 21 | 10 | **6275** |
| **2004** | 4 | 32 | 128 | 939 | 2035 | 2237 | 2364 | 2344 | 1797 | 1310 | 910 | 472 | 160 | 36 | **14768** |
| **2005** | 2 | 8 | 30 | 172 | 476 | 535 | 525 | 854 | 741 | 566 | 390 | 145 | 62 | 12 | **4518** |
| **2006** | 3 | 16 | 184 | 924 | 1739 | 2082 | 2269 | 2490 | 2072 | 1514 | 1004 | 487 | 160 | 28 | **14972** |
| **2007** | 439 | 567 | 522 | 1350 | 3621 | 3729 | 3140 | 3043 | 2491 | 1777 | 992 | 423 | 119 | 22 | **22235** |
| **2008** | 6 | 9 | 45 | 537 | 1049 | 1433 | 1514 | 1754 | 1476 | 1132 | 642 | 395 | 144 | 29 | **10165** |
| **2009** | 19 | 30 | 50 | 73 | 267 | 446 | 432 | 522 | 721 | 604 | 360 | 155 | 51 | 12 | **3742** |
| **2010** | 13 | 37 | 93 | 553 | 1524 | 1691 | 1748 | 1846 | 2001 | 1681 | 867 | 466 | 161 | 48 | **12729** |
| **2011** | 157 | 200 | 525 | 1638 | 4126 | 4798 | 4599 | 3687 | 3377 | 2632 | 1957 | 929 | 253 | 31 | **28909** |
| **2012** | 34 | 43 | 97 | 309 | 1825 | 2319 | 2692 | 2375 | 2357 | 1820 | 1218 | 495 | 193 | 50 | **15827** |
| **2013** | 3 | 32 | 197 | 957 | 2124 | 2819 | 2974 | 2547 | 2426 | 1891 | 1402 | 664 | 172 | 36 | **18244** |
| **2014** | 1 | 5 | 4 | 34 | 156 | 263 | 322 | 379 | 315 | 319 | 173 | 77 | 24 | 6 | **2078** |
| **2015** | 29 | 42 | 43 | 53 | 155 | 283 | 288 | 356 | 325 | 293 | 192 | 105 | 34 | 2 | **2200** |
| **Total** | **867** | **1325** | **2693** | **10001** | **24599** | **28829** | **27796** | **25715** | **22895** | **17953** | **11927** | **5796** | **1861** | **386** | **182643** |

**Table S8 – Female population aged 31-100 for five-year age group, period 1992-2015 (non-aggregated years) in the ATHLOS dataset**

| **Age group** | **31-35** | **36-40** | **41-45** | **46-50** | **51-55** | **56-60** | **61-65** | **66-70** | **71-75** | **76-80** | **81-85** | **86-90** | **91-95** | **96-100** | **Total** |
| --- | --- | --- | --- | --- | --- | --- | --- | --- | --- | --- | --- | --- | --- | --- | --- |
| **Period** |  |  |  |  |  |  |  |  |  |  |  |  |  |  |  |
| **1992** | 36 | 90 | 294 | 968 | 2166 | 2007 | 386 | 89 | 111 | 93 | 86 | 44 | 7 | 3 | **6380** |
| **1993** | 4 | 14 | 46 | 99 | 305 | 222 | 65 | 24 | 46 | 33 | 20 | 11 | 6 | 0 | **895** |
| **1994** | 28 | 74 | 196 | 610 | 1987 | 2122 | 1116 | 53 | 7 | 0 | 0 | 0 | 0 | 0 | **6193** |
| **1995** | 0 | 1 | 1 | 3 | 9 | 3 | 35 | 231 | 789 | 665 | 281 | 142 | 39 | 9 | **2208** |
| **1996** | 13 | 57 | 115 | 408 | 1298 | 2099 | 1776 | 244 | 525 | 506 | 578 | 327 | 106 | 30 | **8082** |
| **1997** | 1 | 1 | 4 | 17 | 33 | 70 | 52 | 4 | 0 | 0 | 0 | 0 | 0 | 0 | **182** |
| **1998** | 21 | 74 | 179 | 584 | 1489 | 2079 | 1928 | 1475 | 1363 | 1234 | 859 | 480 | 174 | 44 | **11983** |
| **1999** | 0 | 2 | 9 | 31 | 73 | 65 | 53 | 58 | 59 | 29 | 17 | 5 | 2 | 0 | **403** |
| **2000** | 350 | 378 | 466 | 745 | 1556 | 2129 | 2186 | 1840 | 1498 | 1403 | 1050 | 598 | 228 | 39 | **14466** |
| **2001** | 123 | 205 | 468 | 1005 | 1814 | 1511 | 1185 | 941 | 622 | 462 | 223 | 111 | 33 | 10 | **8713** |
| **2002** | 14 | 75 | 246 | 741 | 2086 | 2538 | 2887 | 2565 | 1965 | 1641 | 1283 | 685 | 205 | 48 | **16979** |
| **2003** | 35 | 88 | 373 | 1866 | 2787 | 2675 | 2421 | 1880 | 827 | 562 | 328 | 144 | 44 | 13 | **14043** |
| **2004** | 28 | 111 | 407 | 2371 | 4852 | 4799 | 5254 | 5352 | 3833 | 2899 | 2072 | 1041 | 392 | 85 | **33496** |
| **2005** | 8 | 40 | 102 | 426 | 1130 | 1191 | 1146 | 1856 | 1511 | 1115 | 779 | 310 | 127 | 27 | **9768** |
| **2006** | 17 | 71 | 460 | 2044 | 3726 | 4118 | 4230 | 4819 | 3911 | 2808 | 1926 | 1046 | 383 | 69 | **29628** |
| **2007** | 880 | 1078 | 1078 | 2620 | 6771 | 6417 | 5182 | 4789 | 3666 | 2577 | 1467 | 615 | 177 | 36 | **37353** |
| **2008** | 25 | 49 | 136 | 1182 | 2322 | 2982 | 2934 | 3459 | 2896 | 2131 | 1369 | 871 | 328 | 77 | **20761** |
| **2009** | 61 | 90 | 119 | 168 | 634 | 942 | 828 | 1149 | 1701 | 1350 | 835 | 402 | 135 | 36 | **8450** |
| **2010** | 40 | 97 | 226 | 1488 | 3677 | 3908 | 3606 | 3551 | 3827 | 3179 | 1632 | 993 | 348 | 98 | **26670** |
| **2011** | 393 | 490 | 1424 | 3941 | 8171 | 8935 | 8165 | 6443 | 5493 | 4096 | 2833 | 1386 | 409 | 83 | **52262** |
| **2012** | 92 | 139 | 284 | 813 | 4328 | 5211 | 5665 | 4880 | 4656 | 3556 | 2269 | 983 | 404 | 105 | **33385** |
| **2013** | 12 | 89 | 519 | 2217 | 3155 | 4155 | 4056 | 3331 | 3069 | 2326 | 1685 | 801 | 224 | 48 | **25687** |
| **2014** | 2 | 13 | 26 | 104 | 384 | 631 | 721 | 837 | 641 | 591 | 310 | 162 | 55 | 9 | **4486** |
| **2015** | 69 | 96 | 90 | 117 | 333 | 514 | 532 | 601 | 545 | 430 | 276 | 131 | 46 | 5 | **3785** |
| **Total** | **2252** | **3422** | **7268** | **24568** | **55086** | **61323** | **56409** | **50471** | **43561** | **33686** | **22178** | **11288** | **3872** | **874** | **376258** |

**Table S9. Results of the age period cohort models**

| **Priors distributions** | **Males**  **(DIC=650.6)** | **Females**  **(DIC=723.8)** |
| --- | --- | --- |
| Prior and dependence type of the age random effect | Log-gamma (1, 0.00005)  RW2 | Log-gamma (1, 0.00005)  RW2 |
| Prior and dependence type of the period random effect | Log-gamma (1, 0.00005)  RW2 | Log-gamma (1, 0.00005)  RW2 |
| Prior and dependence type of the cohort random effect | Log-gamma (1, 0.00005)  RW2 | Log-gamma (1, 0.00005)  RW2 |
| Prior and dependence type of the overdispersion random effect | Log-gamma (1, 0.005)  IID | Log-gamma (1, 0.005)  IID |
|  |  |  |
|  | **Rate Ratio**  **(mean; median)** | **Rate Ratio**  **(mean; median)** |
| **Age effects (α)** |  |  |
| 31-35 | 0.939  0.937 | 0.851  0.849 |
| 36-40 | 0.925  0.924 | 0.870  0.869 |
| 41-45 | 0.908  0.907 | 0.891  0.890 |
| 46-50 | 0.893  0.892 | 0.914  0.913 |
| 51-55 | 0.890  0.889 | 0.940  0.939 |
| 56-60 | 0.898  0.897 | 0.966  0.965 |
| 61-65 | 0.912  0.912 | 0.991  0.991 |
| 66-70 | 0.934  0.934 | 1.018  1.018 |
| 71-75 | 0.973  0.973 | 1.047  1.046 |
| 76-80 | 1.032  1.031 | 1.076  1.075 |
| 81-85 | 1.100  1.099 | 1.101  1.100 |
| 86-90 | 1.168  1.166 | 1.121  1.120 |
| 91-95 | 1.237  1.235 | 1.136  1.134 |
| 96-100 | 1.307  1.304 | 1.147  1.145 |
|  |  |  |
| **Period effects (β)** |  |  |
| 1991-1995 | 0.615  0.615 | 0.598  0.598 |
| 1996-2000 | 0.738  0.738 | 0.732  0.731 |
| 2001-2005 | 0.921  0.920 | 0.903  0.903 |
| 2006-2010 | 1.074  1.070 | 1.081  1.078 |
| 2011-2015 | 1.184  1.184 | 1.198  1.198 |
| 2016-2020 (prediction) | 1.306  1.305 | 1.328  1.327 |
| 2021-2025 (prediction) | 1.440  1.438 | 1.472  1.471 |
|  |  |  |
| **Cohort effects (γ)** |  |  |
| 1891-1900 | 0.800  0.798 | 0.921  0.919 |
| 1901-1910 | 0.891  0.889 | 0.953  0.950 |
| 1911-1920 | 0.985  0.984 | 0.985  0.984 |
| 1921-1930 | 1.085  1.083 | 1.043  1.042 |
| 1931-1940 | 1.197  1.196 | 1.100  1.100 |
| 1941-1950 | 1.272  1.272 | 1.116  1.116 |
| 1951-1960 | 1.224  1.223 | 1.076  1.076 |
| 1961-1970 | 1.047  1.045 | 1.002  1.001 |
| 1971-1980 | 0.900  0.898 | 0.947  0.945 |
| 1981-1990 (prediction) | 0.772  0.771 | 0.897  0.895 |

**Note.** DIC: Deviance Information Criterion; RW2: Random walk prior of second order. IID: Independent and identically distributed.

**Figure S1. Estimated effects of age, period and cohort in rate ratio terms by gender**


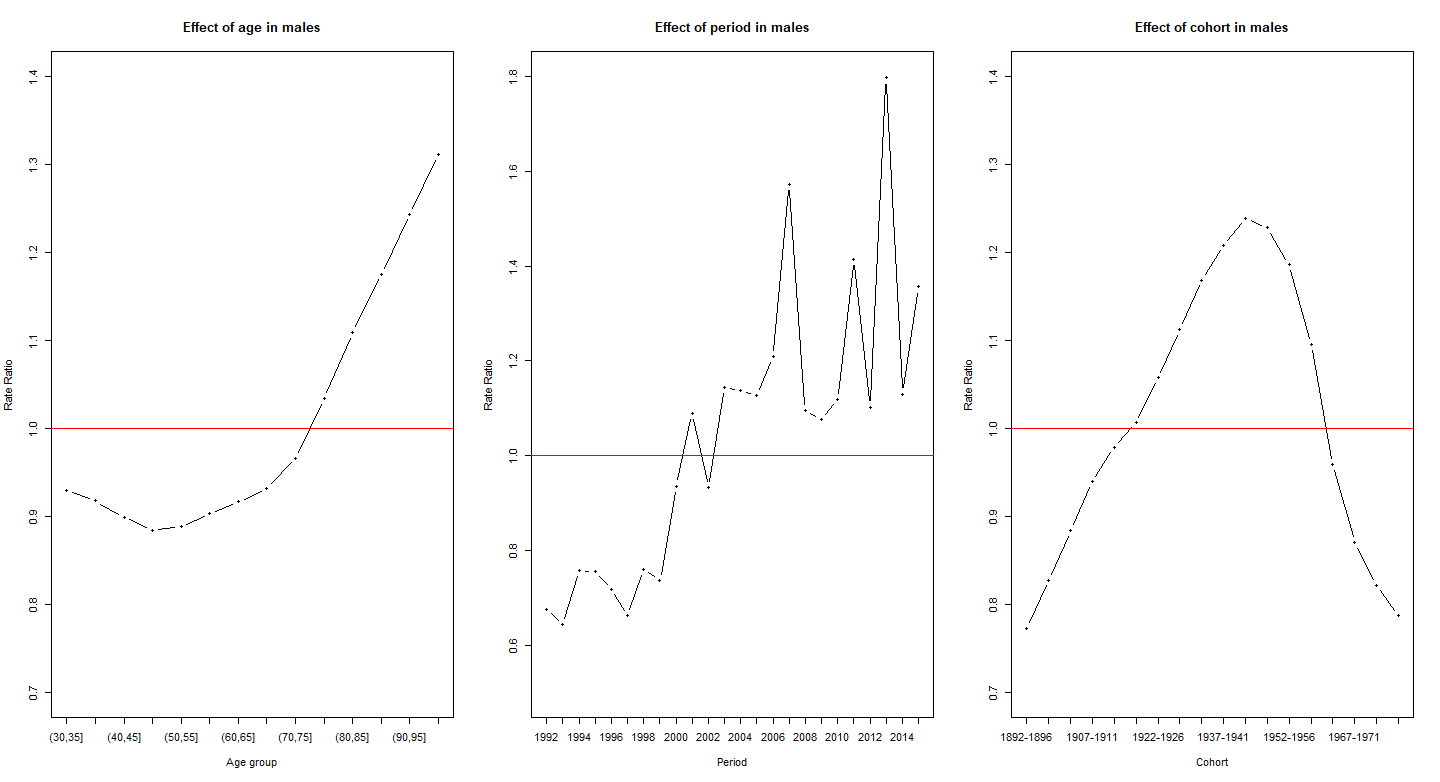


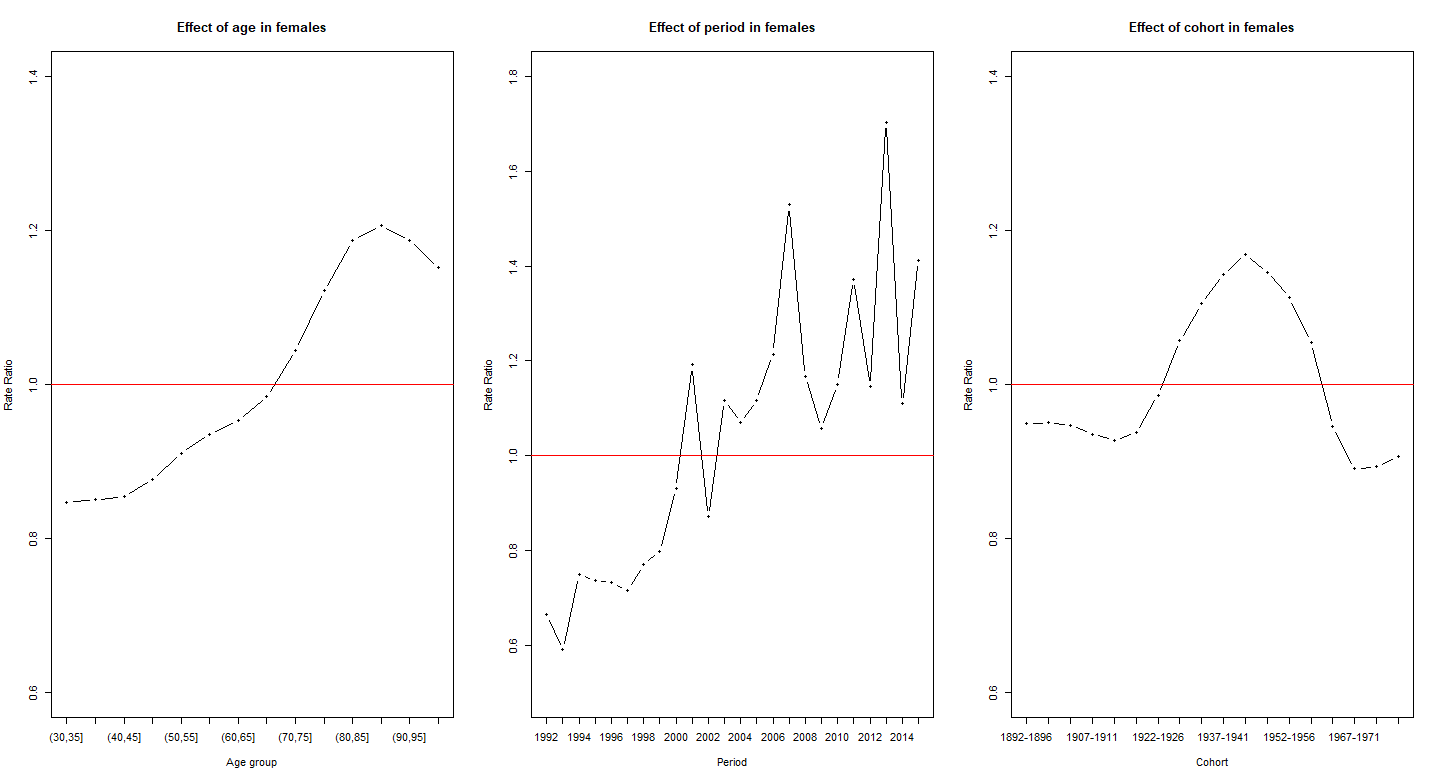


**Note.** The red line indicates rate ratio=1, i.e., the mean effects of age, period and cohort in rate ratio terms are equal to one. In this way age groups, periods and cohorts are comparable to that reference.
